# CD69 Is the Crucial Regulator of Intestinal Inflammation: A New Target Molecule for IBD Treatment?

**DOI:** 10.1155/2015/497056

**Published:** 2015-02-22

**Authors:** Katarina Radulovic, Jan Hendrik Niess

**Affiliations:** ^1^U1019, Team 7, Institut National de la Santé et de la Recherche Médicale (INSERM), 1 rue du Professeur Calmette, 59019 Lille, France; ^2^Center for Infection and Immunity of Lille, Institut Pasteur de Lille, 1 rue du Professeur Calmette, 59019 Lille, France; ^3^Department of Visceral Surgery and Medicine, Inselspital, Freiburgstraße, 3010 Bern, Switzerland

## Abstract

CD69 has been identified as an early activation marker of lymphocytes. However, recent work has indicated that CD69 plays an essential role for the regulation of inflammatory processes. Particularly, CD69 is highly expressed by lymphocytes at mucosal sites being constantly exposed to the intestinal microflora (one of the nature's most complex and most densely populated microbial habitats) and food antigens, while only a small number of circulating leukocytes express this molecule. In this review we will discuss the role of CD69 in mucosal tissue and consider CD69 as a potential target for the development of novel treatments of intestinal inflammation.

## 1. Introduction

CD69 is commonly used as the marker of activated cells, most often lymphocytes and natural killer (NK) cells. But this molecule is much more than a simple activation marker; it is an important regulator of immune responses in the intestine. The primary role of the intestine is absorption of nutrients. Assisting in the digestion and producing essential vitamins and hormones, trillions of commensal bacteria live in the intestinal lumen [1, 2]. The intestinal immune system has to enable the coexistence of these beneficial microorganisms with the host, but also the efficient elimination of pathogens. To achieve these specific tasks, the mucosal immune system of the intestine developed very specific characteristics.

CD69 is highly expressed by lymphocytes at mucosal sites that are separated by a single layer of intestinal epithelial cells from the lumen. Together with the overlying mucus the intestinal epithelium forms a complex and dynamic mucosal barrier that physically prevents the access of luminal bacteria to the deeper sterile tissues [3]. The cells of the mucosal barrier actively participate in the elimination of pathogens by secreting mucus and antimicrobial peptides, presenting microbial derived antigens to T cells, providing tolerogenic signals (mucus proteins) to dendritic cells (DC) and shaping innate and adaptive immune responses by secretion of cytokines and chemokines [4–8].

However, many pathogens are able to avoid these defensive mechanisms and penetrate the mucosal barrier. The complex network of innate and adaptive immune cells underlying the intestinal epithelium is developed to protect host from penetrating pathogens. High proportions of intestinal lymphocytes are effector memory cells to ensure the fast elimination of pathogens that have passed the mucosal barrier [9–11].

On the other side the regulation of overwhelming immune responses to intestinal microorganisms and pathogens is important in the gut to prevent abnormality and tissue destruction that can lead to diseases, such as inflammatory bowel disease (IBD). Regulatory T cells secreting immunosuppressive cytokines, such as transforming growth factor- (TGF-) *β* and interleukin- (IL-) 10, limit overwhelming immune responses to pathogens and are essential for the development of tolerance toward the commensal microflora. Several types of regulatory T cells (Treg) have been described in the gut. Foxp3^+^ Treg are necessary for the development of tolerance in intestine [12] and are the best studied Treg cells. Tr1 and Th3 cells can be induced in oral tolerance models. Tr1 and Th3 cells have regulatory properties depending on the cytokines IL-10 and TGF-*β* [10, 13, 14]. IL-10 and TGF-*β* are also produced by intestinal macrophages and DC, which also contribute to oral tolerance [13, 15].

Both memory T cells and regulatory T cells express CD69 in the gut. In contrast to any other body compartment, intestinal T lymphocytes express high levels of CD69, while only a small number of circulating leukocytes express this molecule in healthy individuals [16, 17]. CD69 is a transmembrane glycoprotein with a C-type lectin domain (CTLD) [18–20]. This molecule is not expressed in detectable levels on naïve leukocytes, but its surface expression is induced promptly upon activation [17–19, 21]. In human diseases, CD69 expression is increased on leukocytes at the site of inflammation [22–25]. Furthermore, early* in vitro* studies described CD69 as a proinflammatory molecule whose engagement with Abs induced intracellular Ca^2+^ influx, lymphocyte proliferation, and the production of proinflammatory mediators, such as IL-2, tumor necrosis factor- (TNF-) *α*, and nitric oxide (NO) [26–30]. CD69 is also necessary for the cell-contact dependent stimulation of macrophages by T cells [31]. However, recent* in vivo* studies with transgenic mice showed that CD69 can limit the immune response and proposed a regulatory function of CD69. CD69 has been shown to have a role in leukocyte migration, in the function of regulatory T cells and resident tissue memory T cells. In contrast to* in vitro* data,* in vivo* studies reported no role of CD69 in the lymphocyte proliferation [32] and T cell priming, therefore excluding the possibility that CD69 serves as a costimulatory molecule [21]. In different murine disease models, including asthma [33, 34], arthritis [35–37], myocarditis [38], pathogen clearance [39], tumor immunity [40, 41], and IBD [42–44], absence of CD69 expression deeply affected the disease course by exacerbating the disease severity in most cases.

Because CD69 is highly expressed by memory T cells and regulatory T cells in the gut, which play an essential role (i) in eliminating pathogens and (ii) in regulating potential harmful immune responses in the gut, we consider CD69 not as a simple activation marker but as a molecule involved in the regulation of immune responses at mucosal sites. We searched http://www.ncbi.nlm.nih.gov/pubmed database (search terms CD69 or inflammation or inflammatory bowel disease) for the studies on CD69 and intestinal inflammation. We found numerous research articles and reviews dealing with the topics of genetic and molecular structure of CD69 and its functional characteristics. In this review we will summarize the current knowledge about the role of CD69 in regulation of mucosal immune system responses in the intestine of mice and humans. Particularly, we will discuss the potential signals driving CD69 expression in the gut, the role of CD69 for the differentiation of regulatory T cells in the gut and review the possible potential of CD69 for the development of novel target therapies for intestinal inflammation.

## 2. How Is the Gene Coding for CD69 Organized and What Is the Molecular Structure of CD69 Protein?

Before we will discuss the relevance of CD69 for the regulation of intestinal immune responses we will briefly summarize the genetic organization of the gene cluster coding for CD69 and the molecular structure of CD69. CD69 (a type II C-lectin transmembrane homodimer protein that consists of disulfide-linked subunits [18–20]) is encoded in the NK gene cluster on chromosome 6 in the mouse and on chromosome 12 in the human genome [19, 22]. When the murine gene locus is compared with the human genome, a 58% homology between them can be identified [17]. The NK gene cluster contains the genes coding for NK cell activating and inhibiting receptors, such as CD94 and NKG2, required for the recognition of the target cells by NK cells. Though being structurally homologous with CD94 and NKG2, CD69 is not involved in target cell recognition by NK cells [17, 19, 45]. Upstream of the transcriptional start site in the mouse* CD69* gene putative binding sites for the inducible transcriptional factors nuclear factor (NF)-*κ*B, erythroblast transformation-specific related gene-1 (ERG-1), and activator protein- (AP-) 1 are located [22].

**Figure 1:**
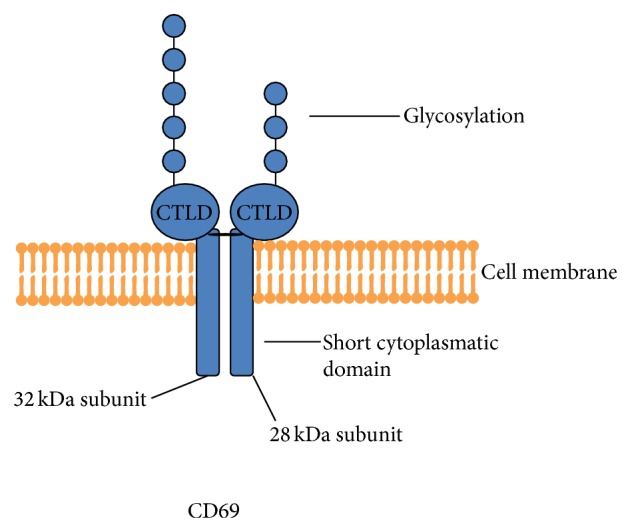
The structure of CD69 molecule. CD69 is membrane-bound protein, a homodimer of two (28 and 32 kDa) differentially glycosylated subunits. Each subunit consists of extracellular C-type lectin domain (CTLD) connected by the short neck region with the single spanning transmembrane domain and short cytoplasmatic tail. Subunits are connected with the disulfide bridge in the extracellular neck region.

The* CD69* gene exists in a single copy. The transcription of* CD69* leads to the formation of 22.5 kDa polypeptide which can be differentially glycosylated to form 28 or 32 kDa subunits (Figure 1) [17]. These subunits can be randomly combined to form 28-28, 28-32, or 32-32 kDa receptors [17, 28, 46, 47]. Each subunit consists of an extracellular CTLD domain connected by the short neck region to the single transmembrane domain and short cytoplasmatic tail (Figure 1) [17, 18, 22, 48]. Subunits are connected with the disulfide bridge in the extracellular neck region (Figure 1) [17].

Because the extracellular subunits of CD69 form a CTLD, it is likely that a yet not identified ligand binds to CD69. Most members of the CTLD family bind bacterial cell surface carbohydrates in a Ca^2+^-dependent manner. Many members of the CTLD family, such as the asialoglycoprotein DC-SIGN, are expressed by macrophages and DC and serve as pattern recognition receptors (PRRs) [17, 49, 50]. The multi-CTLD endocytic receptor CD23 (Fc*ε*RII) is the low affinity receptor for IgE and binds the glycosylated Fc fragment of IgE [51]. CD72 (expressed by B cells) binds the glycoprotein CD5 expressed by T cells, a process required for the costimulation of T cells [52]. Members of the CTLD family hence bind microbial derived cell surface carbohydrates or glycoproteins leading to speculations that CD69 might bind carbohydrates or glycoproteins. When the extracellular domain of CD69 was analyzed by crystallography these studies demonstrated the absence of classical C-type lectin Ca^2+^-binding residues in the extracellular CTLD domain of CD69 [18]. Since classical C-type lectin Ca^2+^-binding residues are missing, carbohydrates are likely not the ligand for CD69 [18, 53]. In order to generate appropriate signal transduction pathways by CD69 both extracellular CD69 CTLD domains are required to be cross-linked indicating that CD69 may rather bind cell associated glycoproteins than soluble molecules as a ligand [17, 36, 40]. Further investigations are needed to identify the physiological ligand of CD69.

**Figure 2:**
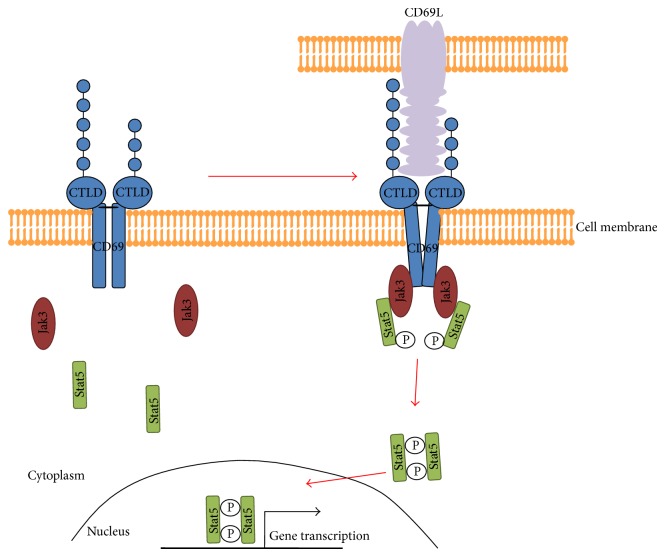
The proposed signalling pathway of CD69. After binding a putative ligand (CD69L) that is most probably a membrane bound protein, the cytoplasmatic tail of CD69 associates with Jak3 kinase. Jak3 recruits and phosphorylates the transcription factor Stat5. Phosphorylated Stat5 (Stat5-P) dimerizes in the active form and translocates to the nucleus where it can regulate the gene transcription.

After cross-linking the extracellular CTLD domains, the cytoplasmatic tail of CD69 generates an intracellular signal transduction pathway [17, 18, 22]. The signaling cascade activated by CD69 is not defined in detail. Recent studies showed that the cytoplasmatic domain of this molecule is associated with the Janus family kinase (Jak)3, which then activates the transcriptional factor STAT5 (Figure 2) [33]. Jak/STAT signaling pathway is evolutionary conserved [54] and regulates central cellular processes, such as development and growth. Its disruption can lead to the development of cancers and/or immune deficiencies. Also, the Jak/STAT signaling pathway regulates immune processes, such as the production of interferons and interleukins. The activation of the Jak3/STAT5 pathway indicates the importance of CD69 for the regulation of cellular processes and the immune system.

## 3. Is CD69 an Early Activation Marker?

Although constantly expressed by monocytes, platelets, Langerhans cells, and a small population of resident lymphocytes in the thymus and secondary lymphoid organs (SLO), CD69 is not found on resting circulating lymphocytes in humans [18, 55–57].* In vitro* cell activation, using various activators, showed rapid induction of CD69 on human T and B lymphocytes, NK cells, macrophages, neutrophiles, and eosinophiles but also on murine T cells and DC [17–19, 21, 55, 56, 58]. Studies in mice showed that* in vivo* type I interferons (IFN-I) strongly upregulate CD69 expression [42, 59, 60]. Furthermore, our group demonstrated that oral administration of a defined antigen to T cell receptor-transgenic mice induces CD69 expression by CD4 T cells in the colonic lamina propria (LP) within 24 h after the feeding [42]. This was not the case with the other activation marker of lymphocytes CD25 [42]; CD25 induction is reported at the late time points after cell activation [61]. Therefore, CD69 is the first activation-induced protein that can be detected on the surface of lymphocytes [61, 62]. Already at 2 h after the stimulation, this receptor can be found on the surface of human lymphocytes, but its expression is transient as it peaks 18–24 h after stimulation and decreases then [17]. One early study on human peripheral blood mononuclear cells (PBMCs) demonstrated that such a rapid induction of CD69 is due to the presence of this molecule in the cytoplasm of resting lymphocytes as its induction was independent of RNA and protein synthesis [58]. This is why CD69 is widely used in studies for the identification of recently activated leukocytes, especially lymphocytes and NK cells, but the role of CD69 in regulating immune processes has not been intensively studied.

## 4. Does the Intestinal Microbiota Regulate the Expression of CD69?

About half of all murine intestinal CD4 T lymphocytes express CD69 in homeostatic conditions [42], indicating their activated state. A constant antigen challenge could lead to high CD69 expression by T cells. Since T cells of the gut are exposed to a high antigen load derived from the intestinal microflora and food the homeostatic balance between inflammatory and immunosuppressive immune processes has been considered as a state of physiological inflammation at mucosal sites [63]. When murine T cells isolated from the gut were compared to T cells isolated from the spleen the proportion of CD69-expressing CD4 T cells was lower in spleen as compared to the gut [42]. When T cells were isolated from the colonic LP of OT-II x RAG^−/−^ mice, a high proportion of CD4 T cells expressed CD69, which was not observed on T cells isolated from the small intestine [42]. CD4 T cells of OT-II x RAG^−/−^ animals kept in specific pathogen-free (SPF) conditions are considered naïve as they specifically recognize chicken ovalbumin (OVA) protein that is not found in food or water of SPF mice facilities. It is surprising that these cells express an activation marker. Possible antigen-independent signals may drive CD69 expression by T cells. Microbial-derived factors recognized by pattern recognition receptors could contribute to the CD69 expression on the surface of colonic T cells [42].

The presence of commensal microorganisms is the crucial factor that contributes to high CD69 expression by intestinal lymphocytes. Reduced surface expression of CD69 by intestinal LP CD4 T cells isolated from germ-free (GF) mice and from mice depleted of intestinal microflora by the treatment with broad-spectrum antibiotics has been reported [42]. In line with our findings, decreased expression of CD69 by intestinal intraepithelial TCR*γδ* ^+^ T cells after the ablation of the microflora has been reported in mice [64]. These results demonstrate that high CD69 expression by lymphocytes in the gut is the consequence of the close proximity of the microflora in intestinal immune compartment. Luminal microorganisms are of importance for the development of mucosal immune responses in the intestine highlighting that the development and function of the mucosal immune system in the intestine differ from the immune system in other body compartments. The induction of CD69 by the specific intestinal environment could play an essential role in shaping immune responses of the gut to protect the host from an uncontrolled invasion of luminal microorganisms. We will hence further discuss the role of CD69 in regulating lymphocyte migration, in controlling the function of resident memory T cells and the differentiation of regulatory T cells.

## 5. Does Lymphocyte Migration Depend on CD69?

CD69 is of importance for the retention of lymphocytes in lymphoid compartments. Activated lymphocytes express CD69, which leads to the retention of lymphocytes in lymph nodes possibly to obtain effector characteristics. Naïve immune cells constantly recirculate through the body, entering the SLO in their search for the potential pathogen-derived antigens and egressing back to the circulation if the specific antigen is not found [65]. The combination of addressins, chemokines, and receptors is tissue-specific to regulate the lymphocyte traffic to a defined tissue compartment. In the intestine, SLO express the chemokines CCL-19 and CCL-21 that bind to CD62L and CCR-7 expressed by naïve-cells, respectively [66–68]. In mice the egress of the lymphocytes from SLO is in general dependent from sphingosine-1 phosphate receptor type 1 (S1P_1_) expression and its interaction with sphingosine-1 phosphate (S1P) from the circulation [59, 69–72]. After egress, lymphocytes that recognized intestine-derived antigens express CCR-9 and *α*4*β*7 that can bind to CCL-25 and MadCAM-1 expressed specifically in the small intestine [66, 67].

Recently, studies in mice pointed at CD69 as one of the major regulators of lymphocyte migration throughout the body. Expressed on activated cells, this molecule captures the lymphocytes that recognized antigen in the SLO for a certain time period that allows them to become fully activated cells [59]. As shown in mice CD69 directly binds S1P_1_ receptor on the lymphocyte surface and mediates internalization of S1P_1_, preventing the lymphocyte egress [59, 70]. Also, CD69 prevents the egress of T cells from the thymus as shown by transgenic overexpression studies in mice [73, 74]. A very recent study demonstrated that CD69 controls selectively the egress of activated antigen-experienced CD4 T cells from Peyer's patches (PP) during the* Salmonella* infection in mice [75]. The same study also showed the existence of CD69/S1P_1_-independent pathway responsible for the global “shut-down” of lymphocyte egress from* Salmonella* infected PP [75]. This creates a need for further investigations of lymphocyte migration during inflammatory conditions in the intestine, as this process could be regulated by completely other molecules than the normal, homeostatic migration. This study also showed a particularly important role CD69 plays in the immune responses of intestinal CD4 T cells [75]. Supporting this, our study showed that CD4 T cell accumulation in the murine colonic LP during IBD is CD69-dependent [43]. Furthermore, the absence of CD69 deeply affected the pattern of chemokine expression and* in vitro* responses to chemokine stimuli by murine CD4 T cells [43]. Hence, CD69 regulates the traffic of intestinal CD4 T cells through complex mechanisms that include both S1P_1_ and chemokines; these processes are of great relevance for the inflammation development in intestine. The importance of CD69 on the lymphocyte migration in human IBD needs to be further investigated.

## 6. Does CD69 Expression Indicate Resident Memory T Cells?

During the immune response the majority of the effector lymphocytes die by apoptosis but a certain number of them remain as long-living memory cells. Memory lymphocytes provide fast and efficient protection during the reexposure to the same antigen again. Different types of memory T cells exist in mice and humans depending on their location and migratory pattern [76]. These types can be distinguished based on the surface markers expression and the cytokine profile. All murine memory cells are expressing high levels of CD44, in contrast to naïve and effector lymphocytes [77, 78], while human memory T cells are usually characterized based on multiple marker expression. Central memory T cells (T_CM_) of humans and mice migrate from the blood to the SLO and express the SLO-homing receptors CD62L and CCR7. T_CM_ cells secrete IL-2, but not effector cytokines [79, 80]. Effector memory T cells (T_EM_) migrate from the blood into the peripheral nonlymphoid tissues as they express the inflamed tissue homing receptors and not CD62L and CCR7. Studies in humans showed that these cells very efficiently protect the peripheral tissues by production of effector cytokines such as IFN-*γ* and IL-4 [79–81]. Recently, the existence of tissue resident memory T cells (T_RM_) has been reported not only in the human skin [82] and lungs [83, 84], but also in murine lungs [83, 84], central nervous system [85], bone marrow (BM) [86], and intestine [87, 88]. In mice CCR7-negative T_RM_ cells are retained in the periphery and do not migrate from the periphery to secondary lymphoid structures [87, 89]. The intestinal T_RM_ cells in mice are particularly well characterized. It is found that the major phenotypic characteristic of these cells is the expression of CD103 and CD69 [87, 89, 90]. TGF-*β* signaling promotes the expression of CD69 and CD103 and therefore is crucial for the formation and maintenance of T_RM_ cells in the murine gut [91]. CD69 is necessary for the formation of BM T_RM_ cells in mice as CD69^−/−^CD4 effector T cells fail to migrate to BM in the late phase of an immune response [92]. Most probably CD69^+^CD49b^+^ effector T cells are the precursors of BM T_RM_ cells as the blockade of their expression impairs the formation of murine T_RM_ CD4 lymphocytes [93]. Very recent study in mice reported the existence of recirculating memory T cells (T_RCM_) that migrate from the peripheral tissues to the local SLO and then further in the systemic circulation [76]. These cells are characterized as CCR7^+^CD62L^int⁡^CD69^−^CD103^+/−^ [76]. This confirms that CD69 is expressed by memory cell subset that is retained in the periphery. Furthermore, studies in humans showed that the expression of CD69 is the major characteristic of the intestinal resident memory T cells and that constant expression of CD69 distinguishes the tissue resident from circulating memory T cells [94]. Hence, CD69 emerges as the major factor that contributes to the immunological memory in the peripheral tissues, such as the intestine. Further studies need to elucidate if CD69 is just a marker indicating T_RM_ cells or if it is involved in regulation of the effector functions (beside retention in lymphoid tissues) of these cells.

## 7. Is the Differentiation of Regulatory T Cells CD69-Dependent?

Treg cells suppress the differentiation and/or proliferation of effector cells, thereby preventing the immune reactions against self-antigens (autoimmunity) and harmless antigens (e.g., commensal microflora). It is considered that Treg cells can be the powerful therapeutic tool for the treatment of inflammatory diseases. Indeed, adoptively transferred Foxp3^+^CD4 Treg cells were able to suppress the disease development in murine models of colitis [95, 96], arthritis [97], and experimental autoimmune encephalitis [98]. Foxp3 Treg cells develop in thymus from positively selected CD69^hi^ TCR^hi^ thymocytes [99]. These cells in both mice and humans can also be generated on the periphery from naïve CD25^−^CD4 T cells in the presence of TGF-*β* and retinoic acid [100–102]. The mechanisms of suppression by Foxp3^+^ cells are largely unknown, but the role of TGF-*β* in this process has been suggested [103]. Our group showed that CD69 affects the generation of murine peripheral Foxp3^+^ Treg cell population as the fraction of these cells was reduced in the intestine of CD69-deficient mice [42]. This effect was especially strong after the oral administration of a specific small protein antigen (OVA) that in normal mice induces the differentiation of Foxp3 Treg cells [42]. Furthermore, naïve CD4 T cells from CD69-deficient animals had a reduced ability to differentiate in Foxp3^+^ cells* in vitro* [42]. Supporting the role of CD69 in the development of Foxp3 Treg cells, several publications reported that crosslinking of CD69 induces the production of TGF-*β* by murine [40, 42, 104] and human cells [105].

Studies on mice and human cells have shown that CD69-expressing CD4 T cells have regulatory properties. In the murine model of spontaneous systemic lupus erythematosus CD69^+^CD4 T cells suppressed the production of proinflammatory cytokines by CD69^−^CD4 T cells [106]. Han et al. in their paper called murine CD69^+^CD4^+^CD25^−^ tumor-induced T cells a new Treg cell subset as they observed suppressive properties of these cells mediated by membrane-bound TGF-*β* [107]. This novel regulatory cell type was also found among human peripheral blood cells and is characterized as CD4^+^LAP/TGF-*β* ^+^Foxp3^−^TGF-*β*RII^+^CD69^+^ cells showing TGF-*β*-dependent suppression of immune responses [108]. These cells accumulate in hepatocellular cancer patients and their number positively correlated with the tumor size [109, 110]. Also, priming the human DC with supernatant of apoptotic tumor cells imprinted the DC to induce CD69^+^ Treg cells [111]. These data confirmed that the presence of Treg cells favors the growth of the cancer. On the other side, high frequency of CD69^+^CD4 Treg cells decreased the risk of graft-versus-host disease after the transplantation of allogenic organs in humans [112].

It is postulated that stable expression of CD69 defines this novel CD4 Treg cell subset. Lymphocyte activation activates the canonical NF*κ*B signaling pathway that controls early and transient expression of CD69 on recently activated human cells [113]. The late and stable expression of CD69 on human lymphocytes is controlled by the noncanonical NF*κ*B pathway [113]. Activation of these different signaling pathways distinguishes activated and regulatory T cells. Confirming this hypothesis, a recent study reported that the anti-inflammatory drug curcumin induced the late phase CD69 expression connected with increased TGF-*β* production* in vitro* [114].

The existence of CD69^+^ Treg cells in intestinal tissues and their possible role in the homeostasis and inflammation in humans has yet to be studied. Oral administration of a defined antigen to mice induced CD69^+^CD4 T cells that are Foxp3-negative but LAP/TGF-*β*1-positive cells in colonic LP [42]. If the Foxp3^−^LAP/TGF-*β*1^+^CD69^+^ cell is a precursor of fully matured peripheral Foxp3^+^ Treg cells needs to be elucidated. To summarize we believe that CD69 regulates TGF-*β* production by T cells and may serve as a regulatory molecule in the immune system. To further discuss our hypothesis we review the role of CD69 in intestinal inflammation.

## 8. Does CD69 Regulate Intestinal Inflammation?

Recent studies in CD69-deficient mice showed that this molecule regulates immune responses in intestine [42–44]. As already discussed, CD69 expression on intestinal lymphocytes is regulated by the microflora. Furthermore, CD69^−/−^ mice were not able to establish oral tolerance to the small food-derived protein OVA [42]. This could be due to the reduced regulatory cell-mediated responses in the absence of CD69. In several different models of experimental colitis, the deficiency of CD69 led to a very serious clinical picture of the disease. Transfer of CD69^−/−^ naïve CD4 T cells into immunodeficient RAG^−/−^ hosts induced a high body weight loss with rise in the systemic levels of proinflammatory cytokines IFN-*γ*, IL-17A, and TNF-*α* as compared to RAG^−/−^ animals receiving T cells from wt animals [42]. In antigen-specific colitis models, the transfer of OVA-specific OT-II CD69^−/−^ naïve CD4 T cells into RAG^−/−^ animals followed by oral delivery of OVA protein resulted also in significant body weight loss and severe colitis [43]. The same was observed in a chemically induced colitis model when dextran sodium sulfate (DSS) was administrated to CD69-deficient mice. These animals develop severe disease with increased transcript levels of the proinflammatory chemokines and cytokines, such as IFN-*γ* [43]. In all these models, histopathological examination of the colonic tissue in mice revealed that absence of CD69 induce increased infiltration of leukocytes and serious damage of the mucosal colonic layer with loss of the Goblet cells and hyperplasia of the crypts [42, 43]. Intriguingly, the recent paper of Hasegawa et al. reported attenuated disease in CD69^−/−^ mice in both acute and chronic DSS colitis models [44]. These contradictions could be the consequence of the different mice background used (B6 and Balb/c), different sources, and the compositions of DSS as well as the different protocols used for the disease induction. Furthermore, different housing conditions in mice facilities, such as the composition of water and food, could induce the alterations in intestinal microflora that can greatly influence the disease development, especially in IBD models.

CD69^−/−^ mice showed increased susceptibility to infection with the food-derived intracellular pathogen* Listeria monocytogenes (Lm)* [39]. Although bacterial clearance capability was the same in wild type and CD69^−/−^ macrophages, increased expression of type I and II IFNs and reduced number of* Lm*-specific T cells were observed in CD69-deficient mice [39]. These mice also showed pathological changes in spleen and liver [39], indicating that they could not control the infection and resolve it locally in the intestine. Furthermore, CD69 affects the disease course in murine models of asthma [33, 34], arthritis [35–37], myocarditis [38], and tumor immunity [40, 41] as demonstrated in CD69-deficient animals. This means that CD69 is not just an activation marker but also strongly involved in the regulation of inflammation.

## 9. Can CD69 Be Targeted for the Treatment of IBD?

We believe that CD69 regulates intestinal inflammation. CD69 is upregulated in patients with Crohn's disease treated with TNF antibodies [115]. How the CD69 pathway could be manipulated for the treatment of patients with IBD will be discussed in the following section. IBD, including Crohn's disease (CD) and ulcerative colitis (UC), is a chronic, progressive, and destructive inflammatory disorder of the gut [11, 116]. This relapsing and remising disease typically occurs in the second or third decade of life and severely affects the patients' quality of life [117]. The disease symptoms include severe diarrhea, rectal bleeding, abdominal pain, fever, weight loss, and fatigue [118, 119]. CD is a patchy and segmental transmural inflammation that can affect any part of the gut, while UC represents the inflammation of mucosal layer that starts at rectum, but it can spread even to the whole colon in the uninterrupted pattern [120]. The progressive bowel damage in CD often leads to the formation of fistulae and granulomas [121]. IBD pathology is very complex and not yet fully understood. Multiple genetic, environmental, and immunological factors that contribute to the disease have been identified [116, 122]. The role of the intestinal microflora in the IBD development is well proven in experiments with mice raised under GF conditions [117, 120]. It is shown that disease severely affects the functions of intestinal epithelial cells inducing the cellular stress accompanied with impaired secretion of mucus and antimicrobial peptides [123, 124]. Both innate and adaptive immune system responses are altered in IBD [116, 120, 125]. In general CD is associated with excessive Th1/Th17 responses, while in UC the elevated levels of Th2- and NK cells-produced cytokines are described [11, 116, 121, 126].

For decades IBD is commonly treated with corticosteroids as an unspecific anti-inflammatory agent [127]. However, the broad palette of side effects and inability of long-term remission phase maintenance by corticosteroid therapy created a need for the novel treatment strategies. Advances in understanding the disease pathology enabled the use of the specific modulators of immune responses. Some of these new modulators affect the effector functions of the immune cells involved in IBD development. For now, the most effective were TNF inhibitors that were able to establish the long-term remission and although they may increase the risk of the opportunistic infections, serious complications are rarely observed [128]. Furthermore, early anti-TNF treatment induced complete mucosal healing [129]. Also anti-IL-6 and IL-6R Abs are showing very promising results in clinical trials [130]. Agents affecting the immune cell migration are also good candidates for IBD treatment. Anti-*α*4 Ab was efficient in the treatment of CD, but it highly increased the susceptibility to the systemic infections [98, 99]. Therefore, anti-*α*4*β*7 Ab that specifically blocks the migration of the lymphocytes to the intestine is tested, proving to be successful in the treatment of UC patients [131]. Anti-CXCL-10 Ab as a cell-specific migration inhibitor that prevents the migration of activated Th1 cells to the periphery is also being tested as a possible treatment for IBD [132]. The blocking agents of CCR9, specific intestinal homing marker, could be beneficial for IBD patients, too. Recent study showed that removal of CCR9^+^ cells by leukapheresis was efficient in IBD treatment, but more extensive studies on this are needed [133].

Most of the studies on the function of CD69 in the diseases are carried out in mice. Sometimes the results obtained from the same disease models are contradictory between different labs showing the need for worldwide standardization in animal breeding conditions and experimental procedures. Also in the context of intestinal immunology, it is known that there are differences between murine and human hut in the microbiota composition and mucosal immune responses. Therefore, studies conducted in mice cannot always be translated to humans. On the other hand, it is difficult to collect all the relevant* in vivo* data in humans. Most of the* in vitro* activation studies on CD69 are done with PBMCs, as there are not many opportunities to isolate cells from the human intestine. Studies in mice showed clearly that CD69 is very important in lymphocyte migration, but whether it has the same role in humans needs to be investigated. Still, the results of human studies on CD69 to date are highly complementary with the data obtained in mice, showing that CD69 has the same expression pattern during homeostasis and inflammatory diseases in mice and humans, being the marker of activated, resident memory or regulatory cells.

Based on the results in studies discussed in this review, the stable induction of CD69 expression should lead to the reduced lymphocyte migration to intestinal LP and to the generation of CD69^+^ Treg cells. It has been shown that T cells isolated from the IBD patients are resistant to TGF-*β* and Treg suppression [134], but the possible role of CD69 in this effect is not known. The exact role of CD69^+^ tissue resident memory cells in intestine should be analyzed in the future studies. Today we are still far away from the possible use of CD69 as a therapeutic agent. Very rigorous and detailed preclinical* in vivo* and* in vitro* studies are required before considering clinical use of CD69-dependent therapy on humans. It has already been observed that targeting a single molecule on lymphocytes can lead to serious complications in humans, while the side effects were absent in all preclinical studies [135]. CD69 targeting can affect the functions of different immune cell types (memory, regulatory and effector lymphocytes) and can modulate the production of both proinflammatory and regulatory cytokines and chemokines. Hence, extensive research on the possible side effects has to be done. Still, CD69 has a profound effect in the functioning of intestinal immune system and this molecule possesses a high potential as a target for the IBD treatment. Identification of the physiological ligand for CD69 receptor would be crucial for the clarification of its role in the immune system and the establishment of the possible therapeutic procedures in the treatment of human diseases.

## 10. Conclusion

**Figure 3:**
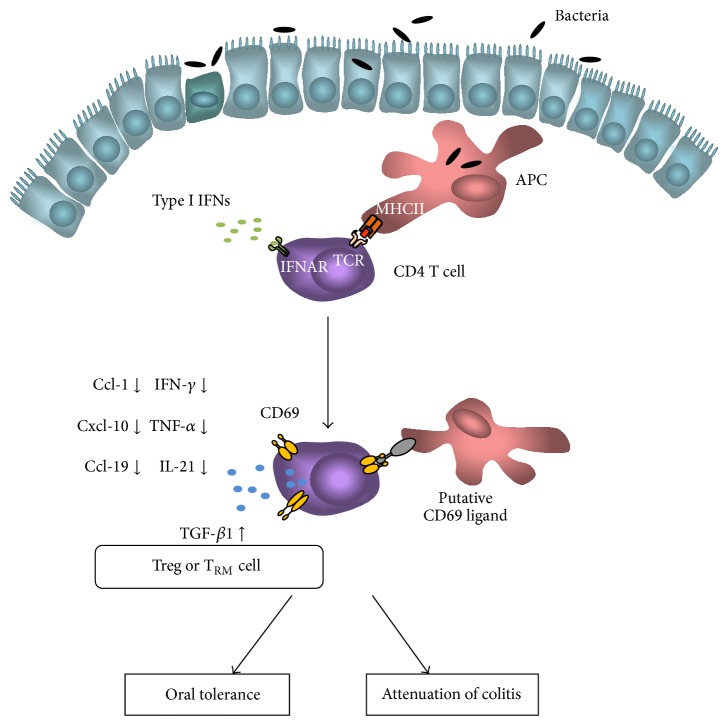
The role of CD69 in mucosal immunity. Activation of intestinal CD4 T cell by antigen recognition, type I interferons (IFN-I), or by presence of intestinal microflora leads to the upregulation of CD69 expression on the cell surface. After binding a ligand, CD69 activates the intracellular pathways that result in decreased production of proinflammatory cytokines (IFN-*γ*, TNF-*α*, and IL-21) and chemokines (Ccl-1, Cxcl-10, and Ccl-19) and increased production of regulatory cytokine TGF-*β*1. If the CD4 T cell establishes a stable expression of CD69, this cell can differentiate into CD69^+^ regulatory T cell (Treg) or tissue resident memory T cell (T_RM_). Therefore, upregulation of CD69 leads to the decreased migration of activated CD4 T cells to the intestine and to the increased regulatory responses, which ensures the establishment of oral tolerance and the attenuation of colitis severity.

CD69 has been for decades used as a simple marker of activated leukocytes without knowing any concrete role this receptor could play in the regulation of immune responses. The discovery that CD69 expression depends on the presence of the intestinal microflora opened new insight into the role CD69 has in immunity and inflammation in intestine. Induced by the specific antigen and/or intestinal microflora, CD69 regulates the essential processes such as the migration of lymphocytes, cytokine secretion, and generation of regulatory and memory T cells at the mucosal sites (Figure 3). CD69 directs the immune responses in the intestine toward the oral tolerance and regulatory responses (Figure 3) [48].* In vivo* CD69 limited the intestinal inflammation proving to be one of the crucial negative regulators of the immune responses in the gut. The activation of CD69 induces tolerogenic cytokines and immune-suppressive cells that could attenuate the inflammation in intestine. Therefore, we believe that CD69 represents a very good target molecule that should be tested for the treatment of IBD.
